# Soft Tissue Scaffolds in Breast Reconstruction: Evolution from Acellular Dermal Matrices to Synthetic Polymers

**DOI:** 10.3390/jcm15093323

**Published:** 2026-04-27

**Authors:** Rebecca Lisk, Thomas J. Sorenson, Carter J. Boyd, Nolan S. Karp

**Affiliations:** Hansjorg Wyss Department of Plastic Surgery, NYU-Langone Health, 222 E 41st Street, New York, NY 10016, USA; rebecca.lisk@nyulangone.org (R.L.);

**Keywords:** soft tissue support, breast reconstruction, acellular dermal matrix, mesh, scaffolds, resorbable

## Abstract

Soft tissue reconstruction often requires biomaterials that provide temporary mechanical support while permitting vascular integration and tissue remodeling. In reconstructive breast surgery, these demands converge within a uniquely challenging environment characterized by large surface areas, variable perfusion, frequent exposure to radiation, and reliance on prosthetic implants. Consequently, breast reconstruction serves as a clinically relevant model for evaluating the performance and limitations of soft tissue scaffolds. Acellular dermal matrices (ADMs) were introduced to provide biologically derived reinforcement capable of host integration and neovascularization. Although ADM has transformed implant-based reconstruction, clinical experience has revealed important limitations, including variability in mechanical properties, inconsistent vascularization, susceptibility to fibrosis, and suboptimal performance in compromised tissue beds. These challenges have driven increasing interest in synthetic polymer scaffolds engineered for reproducible mechanics, controlled degradation, and scalable manufacturing. This narrative review examines the evolution from ADM to synthetic and hybrid scaffold systems in breast reconstruction. We discuss how scaffold architecture, thickness, porosity, and degradation kinetics influence angiogenesis, immune response, and mechanical load transfer during healing. Hybrid strategies that incorporate selective bioactivity within synthetic frameworks are also explored, highlighting their translational promise and current limitations. These principles are particularly relevant in implant-based breast reconstruction, where scaffold performance directly influences complication rates, implant stability, and long-term outcomes. Collectively, breast reconstruction serves as a rigorous translational model demonstrating that optimal soft tissue scaffolds must balance vascular permissiveness, mechanical reliability, and predictable resorption to optimize reconstructive success and guide future biomaterial innovation.

## 1. Introduction

Soft tissue reconstruction often requires coordinated restoration of volume, mechanical support, vascular perfusion, and tissue architecture. In reconstructive breast surgery, these demands converge within a uniquely challenging biological environment characterized by large surface areas, variable perfusion, frequent exposure to radiation, and the presence of prosthetic implants [1,2,3]. As a result, breast reconstruction has emerged as a clinically relevant testing ground for biomaterial-based soft tissue support strategies.

Importantly, the use of soft tissue support devices in breast reconstruction exists within a complex regulatory landscape. In the United States, many acellular dermal matrices and synthetic meshes are not approved by the Food and Drug Administration (FDA) through premarket approval pathways for breast reconstruction [4]. This regulatory gap presents a structural barrier to the generation of high-quality, head-to-head randomized controlled trials, as it complicates study design, reimbursement, and industry sponsorship. Consequently, much of the existing literature consists of retrospective analyses, single-institution series, and heterogeneous comparative studies. These constraints underscore the importance of narrative reviews to synthesize available evidence, contextualize emerging technologies, and identify priorities for future investigation.

Over the past two decades, acellular dermal matrices (ADM) have played a central role in reshaping implant-based breast reconstruction by providing immediate structural support and a biologically derived scaffold for tissue incorporation [5,6,7,8,9]. Their rapid clinical adoption was driven by the promise of host integration, neovascularization, and remodeling into native-like tissue [10,11]. However, accumulating clinical experience has revealed important limitations related to variability, cost, inflammatory response, and inconsistent vascularization, particularly in compromised tissue beds [5,12,13,14,15,16,17,18]. These challenges have catalyzed growing interest in synthetic polymer scaffolds, which, unlike biologic matrices, can be engineered with defined microarchitecture, degradation kinetics, and mechanical properties tailored to the demands of soft tissue support [19,20]. An overview of these materials is found in Table 1.

At its core, the evolution of soft tissue scaffolds in breast reconstruction reflects a fundamental conceptual tension between biologic mimicry and engineering predictability. Early biomaterials, such as acellular dermal matrices, were developed to replicate the structural and biochemical properties of native extracellular matrix, leveraging inherent bioactivity to promote tissue integration and neovascularization. However, variability in biologic materials and dependence on host tissue conditions have underscored limitations in reproducibility and performance. In contrast, synthetic polymer scaffolds are engineered to provide consistent mechanical properties, controlled degradation profiles, and scalable manufacturing. This balance between biological fidelity and engineering precision provides a unifying framework for understanding the progression of scaffold design and informs ongoing innovation in reconstructive biomaterials.

Guided by this framework, this narrative review examines the evolution from acellular dermal matrices to synthetic and hybrid scaffold systems in breast reconstruction, highlighting the biological, mechanical, and translational principles that shape contemporary soft tissue support strategies. The review was conducted in accordance with the Scale for the Assessment of Narrative Review Articles (SANRA). A comprehensive literature search was performed using PubMed/MEDLINE, Scopus, and Google Scholar to identify relevant studies published from January 2000 through January 2026. Articles were selected based on relevance to soft tissue support in breast reconstruction and broader principles of scaffold design in regenerative medicine. Priority was given to systematic reviews, randomized controlled trials, meta-analyses, landmark clinical studies, and influential preclinical investigations. Given the narrative nature of this review, formal risk-of-bias assessment and quantitative synthesis were not performed. Instead, the literature was synthesized to provide a comprehensive and balanced overview of the evolution of soft tissue scaffolds from biologic matrices to synthetic and hybrid materials.

## 2. Acellular Dermal Matrices: Origin and Early Success

Acellular dermal matrices were introduced into reconstructive surgery as a means of harnessing the instructive properties of native extracellular matrix (ECM) while minimizing immunogenicity [21,22]. Derived from human or animal dermis through decellularization processes, ADMs retain key structural proteins such as collagen and elastin, as well as bioactive cues thought to support cell migration, angiogenesis, and tissue remodeling [23,24,25].

In breast reconstruction, ADMs were rapidly adopted to reinforce the implant pocket, define the inframammary fold, and facilitate immediate reconstruction following mastectomy [5,26,27]. Early clinical experience suggested benefits including improved implant positioning, reduced capsular contracture, and enhanced soft tissue coverage [26,28,29,30]. These advantages were attributed to the biologic integration of ADM into host tissue, with neovascularization enabling long-term incorporation [7].

From a bioengineering perspective, ADMs offered an appealing paradigm: a naturally derived scaffold with inherent bioactivity capable of supporting vascular ingrowth without the need for exogenous growth factors or cells [7]. Their success accelerated broader interest in decellularized matrices across soft tissue repair applications, including abdominal wall reconstruction, extremity coverage, and pelvic floor repair [31,32,33].

## 3. Limitations of Acellular Dermal Matrices in Clinical Practice

Despite their widespread adoption, acellular dermal matrices exhibit several limitations that have become increasingly apparent with expanded clinical use and longer-term follow-up. Clinical studies have reported variable complication profiles associated with ADM-assisted breast reconstruction. Reported rates include seroma formation ranging from approximately 5–15% [34,35,36], infection rates of 5–12% [5,37,38], and reconstructive failure rates of 2–8% [37,38], with higher complication rates observed in irradiated or high-risk patient populations. These findings underscore the dependence of biologic matrices on host vascularity and tissue quality. Representative comparative clinical outcomes of acellular dermal matrices and synthetic scaffolds are summarized in Table 2.

### 3.1. Variability in Source, Processing, and Mechanical Properties

ADMs are derived from human or animal dermis and undergo multistep decellularization, sterilization, and preservation processes that vary substantially between manufacturers [25,43,44]. These differences affect collagen architecture, residual bioactive components, thickness, stiffness, and tensile strength [14,15,24,43,44]. As a result, ADM products are not mechanically equivalent, and even within a single product line, batch-to-batch variability may occur [24]. From an engineering standpoint, this lack of reproducibility complicates reliable load sharing and limits precise matching of scaffold mechanics to the evolving demands of healing soft tissue [13].

### 3.2. Inconsistent Vascularization and Host Integration

The clinical utility of ADM relies heavily on timely neovascularization to support incorporation and long-term function. However, vascular ingrowth into ADM is highly dependent on local tissue conditions, including perfusion of mastectomy flaps, prior radiation, and the presence of implants [5,7,12]. In thicker matrices or compromised tissue beds, diffusion limitations and delayed angiogenesis may lead to partial incorporation, persistent acellularity, or chronic inflammatory response [5,12,45,46]. These phenomena challenge the assumption that biologic origin alone guarantees effective vascular integration.

### 3.3. Fibrosis, Inflammation, and Encapsulation

While ADMs are designed to reduce immunogenicity, they are not immunologically inert. Persistent foreign body response, fibroblast activation, and macrophage-mediated inflammation have been observed in both experimental and clinical settings [18,47]. In some cases, this response manifests as excessive fibrosis, thickening of the reconstructed pocket, or a contribution to capsular contracture [17]. Importantly, these outcomes undermine one of the primary theoretical advantages of ADM, which is facilitation of organized, regenerative tissue remodeling rather than fibrosis-dominated scarring.

### 3.4. Performance in Irradiated and Revision Settings

Radiation therapy remains one of the most significant challenges in implant-based breast reconstruction due to its deleterious effects on vascular density, fibroblast function, and tissue compliance [48,49,50,51]. These changes impair wound healing, increase fibrosis, and compromise the biologic environment necessary for scaffold integration. Consequently, irradiated tissues are associated with higher rates of postoperative complications, including infection, seroma, capsular contracture, and reconstructive failure [12,52,53].

Clinical studies have demonstrated that radiation adversely affects both implant-based reconstruction and the incorporation of soft tissue support materials [34,54,55,56]. Histologic and clinical investigations have shown that post-mastectomy radiation therapy (PMRT) disrupts neovascularization and remodeling of acellular dermal matrices, contributing to delayed integration and increased complication rates. Reported outcomes in irradiated prosthetic reconstructions include reconstructive failure rates exceeding those observed in non-irradiated patients, highlighting the vulnerability of biologic scaffolds in compromised tissue beds [38].

Prospective and multicenter studies further underscore these challenges. Investigations such as the iBRA study and subsequent systematic reviews have reported elevated complication rates in irradiated implant-based reconstructions, including higher risks of infection, capsular contracture, and implant loss [5,35,38]. Similarly, meta-analyses evaluating prepectoral reconstruction with PMRT have demonstrated increased risks of adverse outcomes compared with non-irradiated cohorts, but most studies lack sufficient stratification of irradiated subgroups to establish clear superiority of any scaffold category [37,38].

In this context, the limitations of biologically derived matrices have prompted interest in biosynthetic and resorbable polymer scaffolds. Synthetic materials such as poly-4-hydroxybutyrate (P4HB) offer predictable mechanical support and gradual degradation, reducing dependence on host vascularity during the early postoperative period [57]. Clinical and histologic studies have demonstrated favorable integration and remodeling profiles of these materials, supporting their use in high-risk reconstructive scenarios [40,58,59]. While long-term comparative data remain limited, emerging evidence suggests that biosynthetic scaffolds may provide a reliable alternative in irradiated and revision settings.

Revision breast reconstruction presents similar challenges, as prior surgery, fibrosis, and altered vascular anatomy compromise tissue quality and healing potential. In these scenarios, soft tissue support plays a critical role in restoring structural integrity, reinforcing the implant pocket, and optimizing aesthetic outcomes [60]. The need for reliable mechanical reinforcement in compromised tissue beds further underscores the importance of scaffold predictability and controlled degradation. Collectively, these findings highlight the importance of patient-specific biomaterial selection in complex reconstructive settings. Irradiated and revision cases demand scaffolds that balance mechanical stability with vascular permissiveness, reinforcing the rationale for continued innovation and evidence-based application of both biologic and synthetic materials.

### 3.5. Implications for Scaffold Design Evolution

Collectively, these limitations highlight a fundamental tension between biologic mimicry and engineering predictability. While ADMs do offer valuable bioactive cues, their variability, dependence on host vascular conditions, and inconsistent mechanical behavior limit their reliability as universal soft tissue support solutions [13,16]. These shortcomings have driven growing interest in synthetic polymer scaffolds engineered to deliver controlled mechanical support, predictable degradation, and reproducible integration across a broader range of clinical scenarios [34,38].

## 4. Emergence of Synthetic Polymer Scaffolds for Soft Tissue Support

The limitations of acellular dermal matrices have prompted a paradigm shift toward synthetic polymer-based scaffolds. These materials offer reproducible mechanical performance and tunable resorption profiles, enabling scaffold behavior to be aligned with the evolving demands of soft tissue healing [34,38].

Rather than attempting to recapitulate the full biological complexity of native extracellular matrix, synthetic scaffolds are designed around principles of soft tissue repair, that is: temporary load sharing during the initial wound healing phase, followed by gradual transfer of mechanical load to vascularized host tissue [61,62]. In breast surgery, resorbable synthetic scaffolds such as poly-4-hydroxybutyrate (P4HB), Vicryl mesh, and other biosynthetic matrices have been increasingly adopted to provide temporary soft tissue reinforcement while avoiding the long-term persistence of permanent polymers [63,64,65]. These synthetic and biosynthetic meshes have demonstrated comparable clinical outcomes with favorable complication profiles in implant-based breast reconstruction (Table 2). Meta-analyses report infection rates of approximately 3–10% [5,37,38], seroma rates of 3–8% [34,35], and reconstructive failure rates of 1–5% [37,38].

### 4.1. Mechanical Load Sharing

One of the primary advantages of synthetic polymer scaffolds is the ability to define and reproduce mechanical performance parameters such as tensile strength, elasticity, and creep resistance [66,67,68]. In breast reconstruction, these properties influence implant positioning, contour stability, and resistance to deformation under gravitational and muscular forces [68]. Importantly, synthetic scaffolds can be designed to distribute mechanical loads across a broader surface area, reducing focal stress on fragile mastectomy flaps or compromised tissues [35]. This load-sharing function is especially critical in the early postoperative period, when vascular supply is still evolving and tissue tolerance to strain is limited [69].

### 4.2. Host Response and Integration

Unlike biologic matrices, synthetic polymers do not inherently contain bioactive cues. Rather, their integration depends on host-mediated cellular infiltration and neovascularization, and scaffold architecture plays a central role in facilitating this process [70,71]. Properly designed synthetic scaffolds can support fibroblast migration, endothelial ingrowth, and gradual tissue incorporation without excessive fibrosis [71,72,73]. The host response to resorbable polymers is also influenced by degradation byproducts, which must be biocompatible and metabolically managed [74,75]. When degradation kinetics are appropriately matched to tissue healing rates, inflammatory responses are ideally transient rather than chronic, which supports constructive remodeling rather than encapsulation [72,76].

### 4.3. Predictability as Design Motivation

Synthetic polymers offer a level of reproducibility that is difficult to achieve with biologic materials. Polymer chemistry, molecular weight, crystallinity, and processing methods can be precisely controlled, enabling consistent mechanical properties and degradation behavior across batches [42]. This predictability is particularly valuable in breast reconstruction, where scaffold performance must be reliable across patients with widely variable tissue quality, perfusion, and exposure to adjuvant therapies. From an engineering perspective, the goal of synthetic soft tissue scaffolds is not permanent replacement of native tissue, but rather temporary mechanical reinforcement during a biologically vulnerable period [39,41]. By providing early structural support and then resorbing as host tissue remodels, these materials aim to reduce stress concentrations, limit tissue deformation, and promote organized healing [38,40,72].

### 4.4. Resorbable Versus Permanent Polymers

Early generations of synthetic meshes relied on permanent polymers that offered durable strength but were associated with chronic foreign body response, stiffness mismatch, and long-term complications [77]. In contrast, contemporary soft tissue applications increasingly favor resorbable polymers, which are engineered to degrade over months to years in parallel with tissue maturation [41,77,78]. These resorbable scaffolds can be designed particularly so that loss of mechanical integrity coincides with the development of sufficient host tissue strength and vascularization [41,78,79]. This time-dependent design distinguishes modern synthetic approaches from both permanent meshes and biologic matrices, offering a more deliberate alignment between material behavior and wound healing biology [41,78].

## 5. Hybrid and Composite Scaffold Strategies for Breast Soft Tissue Support

As experience with both biologic and synthetic scaffolds has matured, it has become increasingly clear that neither approach alone fully addresses the complex demands of soft tissue reconstruction. This recognition has driven the development of hybrid and composite scaffolds that seek to combine the biological signaling advantages of extracellular matrix-derived materials with the mechanical predictability and scalability of synthetic polymers [80,81].

### 5.1. Rationale for Hybrid Scaffolds

Hybrid scaffolds are motivated by the observation that biologic matrices excel at providing cell-adhesive cues and promoting angiogenesis, while synthetic polymers offer superior control over mechanical behavior, degradation kinetics, and manufacturing consistency [82,83]. By integrating these complementary features, composite systems aim to support early vascularization and tissue integration without sacrificing structural reliability [83]. In breast reconstruction, where scaffolds must function in mechanically loaded, perfusion-limited environments, hybrid strategies offer a theoretical advantage. The goal is not to recreate native tissue wholesale, but to engineer a scaffold that delivers selective bioactivity while maintaining a defined mechanical role during healing [81].

Hybrid scaffold designs span a range of strategies from simple physical composites to more sophisticated biofunctionalized polymers. One approach involves combining a synthetic structural backbone with a biologic surface or coating derived from decellularized matrix components [84]. This configuration preserves bulk mechanical integrity while presenting bioactive cues that facilitate cell attachment and angiogenesis at the tissue–scaffold interface [84]. Another strategy employs biofunctionalization of synthetic polymers, incorporating short peptide sequences, growth factor-binding domains, or extracellular matrix fragments to modulate host response [85,86,87,88]. These modifications can influence macrophage polarization, fibroblast behavior, and endothelial migration without introducing the variability associated with whole-tissue biologics [85,86,87,88].

### 5.2. Hybrid Scaffold Architecture

Beyond material composition, hybrid scaffolds leverage architectural design to enhance vascular integration. Controlled pore size, fiber alignment, and spatial patterning can direct cell infiltration and capillary formation [89,90,91]. In this context, biologic cues function synergistically with physical architecture, reinforcing angiogenic pathways that are already favored by scaffold geometry [91]. For breast soft tissue support, where diffusion distances and tissue thickness are critical constraints, hybrid scaffolds may enable more efficient vascular penetration while maintaining thin, load-sharing constructs [92,93]. This architectural–biological coupling represents a key advance over earlier scaffold designs that relied primarily on material composition alone.

### 5.3. Immunomodulation and Remodeling

An emerging focus of hybrid scaffold development is immunomodulation rather than simple immune avoidance. By shaping the early inflammatory milieu, hybrid materials can promote constructive remodeling rather than fibrotic encapsulation [85,94,95,96]. Selective presentation of bioactive signals within a synthetic framework allows for targeted immune engagement without the prolonged inflammatory responses sometimes associated with biologic matrices [85,94,95,96]. This immunologically informed design approach is especially relevant in breast reconstruction, where chronic inflammation can contribute to capsular contracture, contour irregularities, and reconstructive failure [97].

### 5.4. Translational Status of Hybrid Scaffold Technologies

Although hybrid scaffold technologies offer compelling theoretical advantages, their clinical translation remains heterogeneous (Table 3). Hybrid approaches that incorporate extracellular matrix coatings, peptide functionalization, or growth factor delivery have demonstrated promising results in vitro and in animal models, including enhanced angiogenesis, immunomodulation, and improved cellular integration [85,86,87,88]. However, these technologies are still in preclinical or early investigational stages and have not yet achieved widespread clinical adoption, owing to challenges related to regulatory approval, manufacturing complexity, cost, and the need for long-term outcome data. At present, clinically utilized biosynthetic scaffolds primarily reflect structural hybrids that combine bioresorbable polymers with biologically informed design principles rather than true molecularly biofunctionalized constructs [40,98]. Consequently, most advanced hybrid scaffolds remain investigational, highlighting an important translational gap between biomaterials research and clinical implementation.

### 5.5. Challenges and Limitations

Despite their conceptual appeal, hybrid scaffolds introduce additional complexity in manufacturing, regulatory approval, and clinical deployment [61,99,100]. Combining biologic and synthetic components increases variability, complicates sterilization, and may reintroduce supply chain dependencies [99,100,101]. Furthermore, isolating the clinical benefit of added bioactivity can be challenging, particularly when surgical technique and host factors exert strong influence over outcomes [102]. These considerations underscore the need for indication-specific hybridization, rather than broad application of biologic augmentation. In some clinical scenarios, purely synthetic scaffolds may suffice, whereas in others, targeted biologic signaling may offer incremental benefit [61,84].

## 6. Design Constraints for Soft Tissue Support

Across all soft tissue reconstruction strategies, vascularization remains the principal biological determinant of scaffold success [103,104,105]. Regardless of material composition, mechanical strength, or bioactivity, scaffolds that fail to support timely perfusion are predisposed to inflammation, fibrosis, infection, and structural failure [103]. Breast reconstruction highlights this reality particularly clearly as scaffolds are routinely implanted into tissue beds with compromised vascularity due to mastectomy, prior surgery, or radiation therapy [3].

### 6.1. Diffusion Limits on Scaffold Thickness

Oxygen and nutrient diffusion impose strict physical limits on scaffold design. In the absence of intrinsic vasculature, cell survival within implanted scaffolds depends on diffusion from adjacent tissues until angiogenesis occurs [106]. Excessive scaffold thickness or dense microarchitecture increases diffusion distances, delaying vascular penetration and creating hypoxic regions vulnerable to necrosis and chronic inflammation [106,107,108]. These constraints are especially relevant in breast reconstruction, where scaffolds often span large surface areas and interface with thin, occasionally poorly perfused, mastectomy flaps [84].

Most clinically deployed soft tissue scaffolds rely on angiogenesis with capillary sprouting from existing host vessels [109]. While effective in well-perfused tissues, angiogenesis proceeds more slowly in irradiated or scarred environments [84]. Vasculogenesis offers theoretical advantages but remains challenging to achieve in clinical scaffolds without exogenous cellular or molecular augmentation [110,111]. Synthetic and hybrid scaffold designs increasingly recognize this limitation, emphasizing permissive architecture and degradation rather than attempting to force vascularization through biologic signaling alone [90,112]. This pragmatic approach aligns scaffold function with realistic expectations of host vascular capacity.

### 6.2. Scaffold Architecture and Vascularization

Microarchitectural features such as pore size, interconnectivity, and fiber alignment exert a strong influence over vascular ingrowth [107,112,113,114]. While preclinical investigations frequently define optimal pore sizes and porosity thresholds to promote angiogenesis, these parameters must be interpreted within the constraints of clinically manufacturable scaffolds. Experimental studies suggest that macropores exceeding approximately 100–300 µm facilitate fibroblast migration, capillary ingrowth, and nutrient diffusion, with larger interconnected pores enhancing vascular penetration and tissue integration [78,109,114]. Larger, interconnected pores facilitate endothelial migration and capillary looping, while anisotropic fiber orientation can guide vessel directionality [114]. These architectural parameters may have a greater impact on vascularization than material chemistry alone [112,114].

Commercially available synthetic scaffolds used in breast reconstruction are engineered to approximate these principles through macroporous, knitted architectures [93,107]. Poly-4-hydroxybutyrate (P4HB) scaffolds exhibit a monofilament knitted structure designed to support rapid cellular infiltration, neovascularization, and gradual load transfer during degradation. Histologic and clinical studies have demonstrated progressive host integration and vascularized tissue incorporation within these constructs [57]. Similarly, another is a synthetic, resorbable copolymer mesh composed of fast- and slow-degrading fibers arranged in a porous configuration that provides early mechanical support while permitting tissue ingrowth [39]. Its open-knit architecture promotes fibrovascular integration and aligns with established design principles for soft tissue scaffolds.

Although manufacturers do not always publish standardized pore size metrics comparable to those reported in experimental biomaterials research, the macroporous and interconnected structures of these meshes are consistent with angiogenic design recommendations derived from preclinical studies [66,71,115]. Consequently, currently marketed synthetic scaffolds approximate, rather than precisely replicate, idealized microarchitectural parameters while balancing mechanical strength, handling characteristics, and regulatory requirements. These considerations underscore the importance of translating engineering principles into clinically viable designs. In practice, scaffold effectiveness depends not only on theoretical pore dimensions but also on fiber architecture, degradation kinetics, surgical handling, and host tissue conditions.

### 6.3. Balancing the Timing of Vascularization with Mechanical Support

An underappreciated design challenge is the need to synchronize vascular integration with mechanical load sharing [62,67]. Scaffolds must maintain sufficient strength during the period of limited perfusion while avoiding prolonged persistence that could perpetuate inflammation once vascularization is established [72,79]. Resorbable synthetic polymers are particularly well suited to this temporal coupling, as their degradation profiles can be engineered to align with the progression of angiogenesis and tissue remodeling [72,79,93,116].

### 6.4. Implications for Future Scaffold Design

The experience of breast reconstruction suggests that vascularization should be treated not as a secondary outcome, but as a primary design constraint [103]. Successful soft tissue scaffolds must be thin, porous, and mechanically supportive while remaining permissive to host-driven vascular integration [67,93,114]. Advances in biofabrication, including spatial patterning and gradient architectures, offer promising avenues to further optimize this balance [67,93,117,118].

## 7. Translational Lessons from Breast Reconstruction for Soft Tissue Repair

Breast reconstruction offers a uniquely informative translational model for evaluating soft tissue scaffolds, as it combines high mechanical demand, variable vascularity, frequent use of foreign materials, and long-term functional and aesthetic endpoints [61,84]. Lessons drawn from this field extend beyond breast surgery, informing biomaterial design across diverse soft tissue reconstruction applications [119].

### 7.1. Clinical Context Shapes Biomaterial Performance

One of the clearest lessons from breast reconstruction is that biomaterial performance is inseparable from the surgical context. Tissue perfusion, flap thickness, radiation exposure, and surgical technique exert effects that may overshadow intrinsic material properties [52,69,120]. As a result, scaffolds that perform well in preclinical models or select clinical scenarios may fail when applied indiscriminately [52,121]. This reality argues against one-size-fits-all biomaterial solutions and supports context-specific scaffold selection based on tissue quality and reconstructive goals.

### 7.2. Engineering Reliability Versus Biological Naturality

The historical progression from ADM to synthetic polymers reflects a broader shift away from biological idealism toward engineering reliability. While biologic matrices offer theoretical advantages in bioactivity, their dependence on host vascular capacity and inherent variability limit reproducibility [121,122]. Synthetic scaffolds, by contrast, prioritize consistent mechanical behavior and controlled interaction with host biology [37]. Breast reconstruction demonstrates that predictability and controlled degradation can be as clinically valuable as biologic mimicry, particularly in hostile tissue environments [37,40,53].

### 7.3. Surgical Technique Is a Determinant of Scaffold Success

Scaffold performance cannot be fully separated from surgical handling, fixation, and placement. Tension distribution, scaffold orientation, and interface with native tissue all influence mechanical load transfer and vascular access [35,98,103,123]. Breast reconstruction highlights the importance of surgeon–engineer alignment, where material design anticipates real-world surgical use rather than idealized deployment conditions. Complications such as infection, seroma, capsular contracture, and reconstructive failure provide critical feedback for scaffold design [36,124,125,126,127]. These failure modes often reflect mismatches between scaffold properties and tissue biology, including excessive stiffness, delayed resorption, or inadequate vascular integration [62,98]. Incorporating clinical failure data into iterative design cycles is essential for advancing soft tissue scaffold technology [115].

### 7.4. Broader Implications for Soft Tissue Reconstruction

The evolution of scaffold use in breast reconstruction illustrates a generalizable framework for soft tissue repair: prioritize vascular permissiveness, match mechanical support to healing timelines, and design for reproducibility and scalability [61,93,99,103]. These principles apply across reconstructive contexts, from abdominal wall repair to extremity reconstruction and beyond [46,89]. By serving as a clinically demanding testbed, breast reconstruction continues to inform the development of next-generation biomaterials that integrate engineering precision with biological realism [119].

### 7.5. Context-Dependent Scaffold Selection

Scaffold performance in breast reconstruction is inherently context-dependent. Clinical outcomes are influenced not only by material composition and microarchitecture but also by surgical approach, tissue quality, and patient-specific risk factors (Table 4). Immediate reconstruction, for example, often involves thin and variably perfused mastectomy flaps, necessitating scaffolds that provide reliable mechanical support while permitting rapid vascular integration. In contrast, delayed reconstruction typically occurs in more mature but frequently fibrotic tissue beds, where reinforcement and contour stability may be prioritized.

The choice between prepectoral and subpectoral reconstruction further illustrates this principle. Prepectoral implant placement relies heavily on soft tissue support to ensure implant coverage and stability, thereby increasing dependence on scaffold performance. Subpectoral reconstruction, while providing muscular coverage, introduces dynamic forces that influence implant position and mechanical loading, altering the functional role of adjunctive materials. Similarly, irradiated tissues present unique biological challenges characterized by reduced vascularity, impaired wound healing, and increased fibrosis. These factors significantly influence scaffold integration and complication risk, distinguishing them from non-irradiated settings. Revision cases introduce additional complexity due to prior scarring, altered anatomy, and compromised perfusion, necessitating materials with predictable mechanical properties and favorable host responses. Collectively, these considerations underscore that no single scaffold is universally optimal. Instead, successful outcomes depend on aligning biomaterial properties with the biological and mechanical realities of each clinical scenario.

## 8. Economic Considerations: Cost, Reimbursement, and Accessibility

Beyond biological performance and mechanical reliability, cost and accessibility significantly influence the adoption of soft tissue scaffolds in breast reconstruction. Acellular dermal matrices (ADMs), while widely used, are associated with substantial expense and variability in pricing due to differences in sourcing, processing, and distribution [38,40,128,129]. Their rapid integration into reconstructive practice was facilitated in part by favorable reimbursement structures, which supported their widespread clinical adoption despite limited early high-level comparative data [128].

Synthetic and biosynthetic scaffolds have emerged as potential alternatives with more standardized manufacturing processes and scalable production. These features offer theoretical advantages in supply chain reliability and cost predictability. However, these contemporary resorbable polymers carry meaningful costs, reflecting the complexity of advanced biomaterial engineering and regulatory compliance [40,130]. Consequently, synthetic scaffolds should not be viewed as inherently less expensive than ADMs but rather as offering distinct economic and performance trade-offs.

Comparative economic analyses suggest that cost-effectiveness depends on multiple factors, including complication rates, reoperation risk, operative efficiency, and long-term reconstructive outcomes [37,38,130]. Reductions in complications such as infection, seroma, or implant loss may offset higher upfront material costs, emphasizing the importance of value-based evaluation rather than simple cost comparison [65]. In this context, scaffold selection should be individualized based on patient risk profiles, institutional resources, and payer environments.

Accessibility also varies across healthcare systems and geographic regions, where reimbursement policies, regulatory pathways, and resource availability influence material utilization. As next-generation synthetic and hybrid scaffolds continue to evolve, demonstrating both clinical efficacy and economic value will be essential to ensure equitable access and sustainable integration into reconstructive practice.

## 9. Future Directions in Soft Tissue Scaffold Design

Continued progress in soft tissue reconstruction will depend on scaffold strategies that integrate vascular permissiveness, mechanical reliability, and immunologic control while remaining practical for surgical translation [76,90,103,131,132]. Insights gained from the evolution of scaffold use in breast reconstruction suggest several priority directions for future biomaterial development.

### 9.1. Vascular-Patterned and Gradient Scaffolds

One promising avenue is the incorporation of spatially defined architectures that actively guide vascular ingrowth [118,133,134,135,136]. Advances in biofabrication, including additive manufacturing and controlled fiber deposition, enable the creation of scaffolds with gradient porosity, anisotropic mechanics, and preferential vascular pathways [137,138,139,140]. Such designs may better accommodate diffusion limits while preserving load-sharing capacity, particularly in large or perfusion-limited soft tissue reconstructions [118,141]. Rather than relying solely on biochemical cues, these approaches emphasize physical guidance of angiogenesis, aligning scaffold geometry with known principles of vessel formation and remodeling.

### 9.2. Immunomodulatory Material Design

Future scaffolds are likely to move beyond passive biocompatibility toward active modulation of the host immune response [132,142]. Early inflammatory signaling plays a decisive role in determining whether healing proceeds toward regenerative remodeling or fibrotic encapsulation [132]. Synthetic and hybrid scaffolds that bias macrophage polarization and fibroblast behavior toward constructive pathways may reduce long-term complications such as fibrosis and contracture [85,86,132]. Importantly, immunomodulation must be tightly controlled and indication-specific to avoid unintended systemic or chronic effects [143].

### 9.3. Personalized and Context-Specific Scaffold Selection

As understanding of tissue-specific biology improves, scaffold selection is likely to become more personalized, accounting for factors such as prior radiation, tissue thickness, vascular status, and reconstructive goals [98,103,121,144]. Rather than a single “best” scaffold, future practice may involve algorithmic or decision-guided material selection, matching scaffold properties to patient-specific risk profiles [145,146]. Breast reconstruction, with its heterogeneity of clinical scenarios, provides a natural framework for developing and validating such precision approaches.

### 9.4. Minimally Invasive and Injectable Strategies

Injectable and minimally invasive scaffold systems represent an emerging frontier in soft tissue support [147]. These approaches aim to reduce surgical trauma while providing localized mechanical reinforcement and biological modulation [147]. While challenges remain in achieving sufficient mechanical strength and spatial control, advances in shear-thinning hydrogels and in situ polymerization may expand the applicability of these systems [148,149,150].

### 9.5. Regulatory Constraints and Implications for Evidence Generation

The absence of FDA premarket approval for soft tissue support devices in breast reconstruction represents a significant structural limitation in the current evidence landscape [4]. As previously described, this regulatory environment complicates the design and execution of prospective randomized trials, particularly those comparing biologic and synthetic scaffolds. Without formal regulatory indications, investigators face barriers related to funding, trial standardization, and ethical considerations, which collectively hinder the development of high-level comparative evidence. As a result, much of the existing literature is derived from retrospective studies, institutional experiences, and systematic reviews of heterogeneous data. This constraint informs the rationale for the narrative approach adopted in the present review, which aims to synthesize mechanistic insights, clinical outcomes, and engineering principles in the absence of robust randomized data. Recognition of this regulatory gap is essential when interpreting current evidence and underscores the need for future collaborative efforts among clinicians, researchers, industry stakeholders, and regulatory agencies to facilitate future trials.

### 9.6. Translational Integration

Finally, future success will depend on tighter integration between material scientists, surgeons, and clinical researchers. Iterative design informed by real-world failure modes will be essential [115,151]. Breast reconstruction offers a valuable feedback-rich environment for this process, accelerating translation from bench to bedside.

## 10. Conclusions

The evolution of soft tissue scaffolds in breast reconstruction from acellular dermal matrices to synthetic polymers and hybrid systems reflects a broader maturation of biomaterial design philosophy. Early reliance on biologic mimicry has given way to a more pragmatic emphasis on engineering predictability, vascular permissiveness, and controlled host interaction. Clinical experience has demonstrated that successful soft tissue support depends less on material origin than on alignment between scaffold properties and the biological and mechanical realities of the reconstructive environment. Breast reconstruction, with its high demands and frequent vascular compromise, serves as a stringent translational model for evaluating these principles. As the field advances, future scaffold strategies will need to prioritize vascular integration as a primary design constraint and couple mechanical support to healing timelines, while accommodating changing and imperfect patient-specific tissue conditions.

## Figures and Tables

**Table 1:** Representative Soft Tissue Scaffolds Used in Breast Reconstruction.

Scaffold Category	Representative Materials	Commercial Examples	Key Design Features	Advantages	Limitations
Acellular Dermal Matrices (ADM)	Decellularized human or animal dermis retaining extracellular matrix proteins (collagen, elastin, structural ECM)	AlloDerm^®^, DermACELL^®^, FlexHD^®^, SurgiMend^®^, Strattice^®^	Biologic scaffold intended to support host cell infiltration and neovascularization	Biologic integration Good handling characteristics Extensive clinical experience	Batch variability High cost Inconsistent vascularization Risk of inflammatory response or fibrosis
Synthetic Permanent Meshes	Non-resorbable polymers (e.g., polypropylene, polyester)	Prolene^®^ mesh, Parietex^®^ mesh	Durable polymer mesh providing long-term mechanical reinforcement	Strong mechanical support Low material variability	Chronic foreign body response Stiffness mismatch Long-term complications Limited use in contemporary breast reconstruction
Synthetic Resorbable Scaffolds	Bioabsorbable polymers designed to degrade over time (e.g., poly-4-hydroxybutyrate, polylactic acid, polyglactin)	GalaFLEX^®^ (P4HB), TIGR^®^ Matrix, Vicryl^®^ mesh	Temporary load-sharing scaffold that gradually transfers mechanical support to host tissue	Predictable mechanical properties Controlled degradation Lower long-term foreign body burden	Limited bioactivity Dependence on host vascularization Long-term outcome data still evolving
Hybrid/Composite Scaffolds	Combination of synthetic polymers with biologic components or biofunctionalized surfaces	P4HB with ECM coatings (experimental), collagen-polymer composites, biofunctionalized electrospun scaffolds	Integrate mechanical reliability of synthetic materials with selective biologic signaling	Potential for improved angiogenesis Potential for improved immune modulation Tunable architecture	Increased manufacturing complexity Regulatory challenges Limited clinical adoption to date

Key: ECM extracellular matrix, P4HB poly-4-hydroxybutyrate, ADM acellular dermal matrix. AlloDerm (Allergan Aesthetics, Dublin, Ireland), DermACELL (LifeNet Health, Virginia Beach, VA, USA), FlexHD (MTFBiologics, Edison, NJ, USA), SurgiMend (Integra Life Sciences, Princeton, NJ, USA), Strattice (Allergan Aesthetics, Dublin, Ireland), Prolene (J&J MedTech, New Brunswick, NJ, USA), Parietex (Medtronic, Galway, Ireland), GalaFLEX (BD, Lexington, MA, USA), TIGR (Novus Scientific, Uppsala, Sweden), Vicryl (J&J MedTech, New Brunswick, NJ, USA).

**Table 2:** Representative Clinical Outcomes of Acellular Dermal Matrices and Synthetic Scaffolds in Implant-Based Breast Reconstruction.

Outcome	Acellular Dermal Matrix (ADM)	Synthetic/Biosynthetic Scaffolds	Key References
Infection	5–12%	3–10%	Dikmans et al. [5]; Makarewicz et al. [37]; Arnautovic et al. [38]
Seroma	5–15%	3–8%	iBRA Study [35]; Faulkner et al. [34]
Capsular Contracture	Reduced relative to submuscular techniques; variable incidence	Comparable or low rates reported	Becker et al. [39]; Levy et al. [40]
Reconstructive Failure	2–8%	1–5%	Makarewicz et al. [37]; Arnautovic et al. [38]
Explantation	2–6%	1–4%	Faulkner et al. [34]; Levy et al. [40]
Patient-Reported Outcomes	High satisfaction reported in select studies	Limited but comparable early data	Woussen et al. [6]; iBRA Study [35]
Mechanical Predictability	Variable due to biologic origin	Highly reproducible	Panayi & Orgill [41]; Janouskova [42]
Cost and Manufacturing	High and variable	More scalable and consistent	Makarewicz et al. [37]; Becker et al. [39]

**Table 3:** Translational Status of Hybrid Scaffold Strategies in Soft Tissue Reconstruction.

Hybrid Strategy	Representative Examples	Mechanism	Stage of Translation	Clinical Relevance
ECM-Coated Synthetic Polymers	Decellularized matrix–coated meshes	Enhances cell adhesion and angiogenesis	Preclinical/Experimental	Investigational
Peptide-Functionalized Polymers	RGD-modified scaffolds	Promotes cell attachment and integration	Preclinical	Investigational
Growth Factor–Incorporated Scaffolds	VEGF- or FGF-loaded constructs	Stimulates angiogenesis	Preclinical/Early Translational	Investigational
Biohybrid Polymer–ECM Composites	Collagen–polymer composites	Combines bioactivity with mechanical strength	Preclinical to Early Clinical	Limited clinical data
Biosynthetic Resorbable Meshes	Poly-4-hydroxybutyrate (P4HB)–based scaffolds	Temporary load-sharing with host integration	Clinical Use	Widely adopted in breast surgery
ECM-Inspired Synthetic Scaffolds	Biomimetic polymer architectures	Mimic extracellular matrix structure	Early Translational	Emerging technologies

Key: ECM: Extracellular matrix; P4HB: Poly-4-hydroxybutyrate.

**Table 4:** Clinical Contexts Influencing Scaffold Selection in Breast Surgery.

Clinical Context		Key Considerations	Implications for Scaffold Selection
Type of Reconstruction	Immediate	Thin mastectomy flaps, evolving perfusion	Emphasis on vascular permissiveness and mechanical support
	Delayed	Fibrosis, prior surgery, and radiation	Requires durable reinforcement and predictable integration
Plane of Reconstruction	Prepectoral	Reliance on soft tissue support for implant coverage	Increased dependence on scaffold strength and stability
	Subpectoral	Muscular coverage and dynamic forces	Reduced reliance on scaffolds but altered mechanical loading
Radiation Status	Irradiated Fields	Reduced vascularity, increased fibrosis	Higher complication risk; materials must support integration in compromised tissues
	Non-Irradiated Fields	Improved healing environment	Broader range of viable biomaterials

## Data Availability

No new data were created or analyzed in this study.
